# Hip arthroscopy after periacetabular osteotomy for acetabular dysplasia – incidence and clinical outcome

**DOI:** 10.1186/s12891-022-05625-x

**Published:** 2022-07-12

**Authors:** Pierre Laboudie, Thomas Dymond, Cheryl Kreviazuk, George Grammatopoulos, Paul E. Beaulé

**Affiliations:** 1 Division of Orthopaedic Surgery, The Ottawa Hospital (TOH), General Campus, 501 Smyth Road, CCW 1640, Ottawa, ON K1H 8L6 Canada; 2 Faculty of Medicine, The University of Ottawa, Ottawa, ON Canada; 3 Orthopaedic surgery department, Cochin hospital, Paris, France; 4 Clinical Epidemiology Program, The Ottawa Hospital Research Institute, Ottawa, ON Canada

**Keywords:** Hip, Acetabular dysplasia, Periacetabular osteotomy, Hip arthroscopy

## Abstract

**Background:**

The periacetabular osteotomy (PAO) is the treatment of choice for acetabular dysplasia and has demonstrated improvement in patient reported outcomes measures (PROMs) as well as acceptable long-term survival. However, acetabular dysplasia is also associated with intra-articular lesions that can negatively impact clinical outcome. This study aimed to analyse the incidence, operative findings, and outcomes of hip arthroscopy after PAO.

**Methods:**

This is a single center retrospective study by querying our hip preservation prospectively collected database from 2006 to 2020. All patients having undergone hip arthroscopy after a PAO, with a minimal follow-up of one year, were identified. 202 PAOs were done with a mean age of 28.3 years (12.7 – 53.6) including 39 males and 167 females. Failure was defined as conversion to hip replacement. Demographics, surgical findings, reoperations, and PROMs (pre and post operatively at the last follow-up point only for hips not converted to hip replacement).

**Results:**

Fifteen hips in 15 patients (7.4%) out of 202 PAOs underwent a hip arthroscopy at a mean time of 3.9 years (0.3–10.3) after PAO. There were 2 males, 13 females and the mean age was 29.8 years (18.5–45). 12 hips had no radiological osteoarthritis (Tönnis 0) and 3 hips had early osteoarthritis (Tönnis 1). At time of arthroscopy, all hips had a labral tear, 9 had a chondral damage ≥ Beck 4. Eight hips had labral debridement, 7 had labral repair, 2 had resection of adhesions and 4 underwent a femoral osteochondroplasty. Four hips (27%) were converted to a hip replacement at a mean time of 1.8 years(0.5–3.2) after hip arthroscopy. Patients converted to hip replacement were significantly older (*p* = 0.01), had a lower post-PAO LCEA (*p* = 0.01) and a higher post-PAO Tönnis angle (*p* = 0.02). There were no significant improvements in PROMs.

**Conclusion:**

This study reports a hip arthroscopy reoperation rate after PAO of 7.4%. All three types of dysplasia (uncovered anteriorly, posteriorly, or globally) were present in this cohort. Twenty seven percent of patients were converted to hip replacement and PROMs were not significantly improved by hip arthroscopy. Therefore, this procedure should be approached with some caution.

## Introduction

The long-term survival of periacetabular osteotomy(PAO) for acetabular dysplasia [1, 2] has been shown to be between 60 and 74% twenty years after surgery [3–5]. However, as much as 11% of patients [6] continue to experience symptoms after PAO alone. One of the probable causes is concomitant intra-articular pathology which is reported to range from 60–85% of patients have concomitant intraarticular pathology (cartilage damage) [7, 8]. Other leading factors for failure are higher age and preoperative osteoarthritis ​degree [3, 4, 9]. This has led some to include arthroscopy at the time of the PAO, with acceptable results and complication rates [10]. However, the results of hip arthroscopy for persistent symptoms after isolated PAO for acetabular dysplasia are not well known [6].

This study aimed to analyse the incidence, the operative findings and outcomes of hip arthroscopy after PAO. Our hypothesis was that hip arthroscopy after isolated PAO could be beneficial in selected patients.

## Methods

### Study design and population

This is a single-center, retrospective study of a prospective data base, Institutional review Board (IRB) approved cohort study. Our prospective hip preservation surgery database was queried to identify a series of patients who underwent hip arthroscopy for recurrent hip pain following previous PAO performed for acetabular dysplasia between 2006 and 2020 by two surgeons and with a minimum follow-up of one year. Patients who underwent a combined PAO and hip arthroscopy/arthrotomy were excluded of the study. PAO was performed with a previously described technique [9]. The hip arthroscopy was performed by the surgeon who performed the index procedure, with the patient positioned supine on a traction table, with a central compartment access first and with an interportal capsulotomy and without capsular closure. Indications to perform hip arthroscopy were recurrent pain, mechanical symptoms such as catching or subjective instability as well as labral pathology according to an MRI (Magnetic Resonance Imaging). All patients had a positive diagnostic intra-articular anesthetic injection.

### Outcomes measures

All outcomes of interest were prospectively recorded. Length of outcome was determined from the last clinical encountered. Demographics characteristics were collected (gender, body mass index (BMI), age at the time of PAO and of hip arthroscopy, previous ipsilateral hip surgeries). Standardized radiographic evaluation was performed pre and post PAO with analyze of the lateral center edge angle(LCEA) and the Tönnis angle or acetabular index (AI), both measured at the most lateral point of the acetabular sourcil [11, 12]. Pre and post alpha angles were measured on the Dunn View [13]. Radiological osteoarthritis at the time of hip arthroscopy was classified according to the Tönnis classification [14]. The initial acetabular dysplasia was diagnosed and classified according to the Ottawa classification [15] whose reliability has been demonstrated [16]. Surgical findings and procedures at the time of the hip arthroscopy were collected (labral tear, labral procedure, chondral damage as per Beck [17], post-operative adhesions, femoral osteochondroplasty(FOCP) for cam lesion). Reoperations after the hip arthroscopy were collected and conversion to hip replacement was defined as failure. Predictors of failures were tested for association. Pre and post operative patients reported outcomes measures (PROMs) were collected: the WOMAC score[18](pain, stiffness and function), the HOOS [19] (pain, symptoms, activities of daily living, sports and quality of life), the UCLA [20] score and the SF-12 [21] mental and physical score. The minimal clinically important difference (MCID) and the patient acceptable symptom state (PASS) were determined for the HOOS subscales. The MCID values were 5 points for HOOS-ADL and 6 points for HOOS-SRA. Furthermore, we determined PASS values as described by Chahal [22] as 87 for the HOOS-ADL and 75 for the HOOS-SRA. Post operative PROMs were collected at the last follow-up point for the hips not converted to hip replacement.

### Statistical analysis

Data were summarised using descriptive statistics including count and percentages for categorical variables. Continuous variables were described using the mean, minimum and maximum. Categorical variables were presented with total count and percentages. The Chi-squared and Fisher’s exact tests were used to test for differences between categorical variables and the The Wilcoxon test was used for continuous variables. All analysis was performed using IBM SPSS (Statistical Product and Service Solutions) software for Windows (version 27).

## Results

### Demographics

**Fig. 1:**
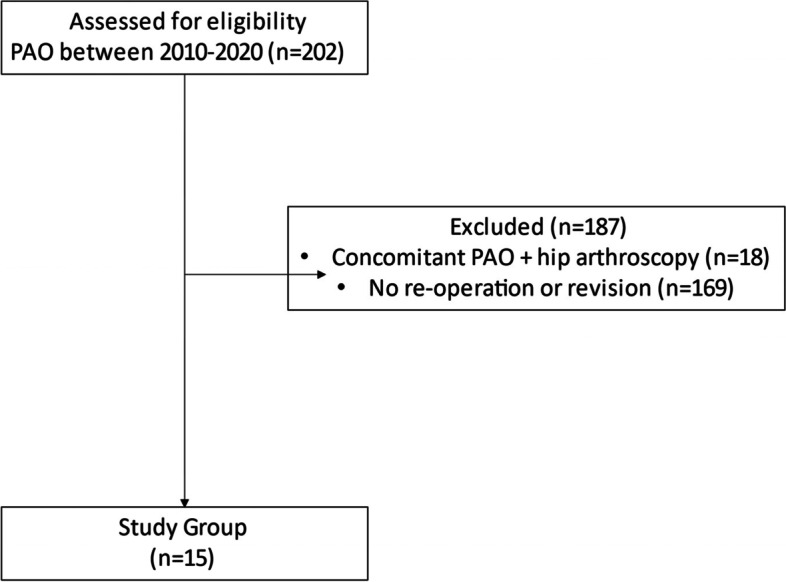
Flowchart of the patients

Of 202 PAO patients, 15 hips in 15 patients (7.4%) underwent hip arthroscopy (Fig. 1) for persistent pain and poor function with 2 males and 13 females. The mean follow-up was 4.6 years (1 – 12) after hip arthroscopy and 8.4 years (1.5 – 15) after PAO. Demographics are summarized in Table 1.

**Table 1:** Demographics of the cohort

Demographics	
Gender	2 males, 13 females
Age at PAO (years); median(range)	24 (16 – 40)
Age at hip arthroscopy (years); median(range)	29(18.5 – 45)
Time from PAO to hip arthroscopy (years); median(range)	2.5 (0.3 – 10.3)
BMI (kg/m^2^); median(range)	25 (20 – 42)
Previous ipsilateral hip surgery	4(27%): 3 hip arthroscopies, one pelvic (Salter) osteotomy

The 3 types of dysplasia according to the Ottawa classification were found with one anterior dysplasia, 5 posterior dysplasia and 9 global dysplasia. Radiographic details are summarized in Table 2.

**Table 2:** Radiographic findings of the cohort

Radiographic details	
Tönnis classification	12 grade 0, 3 grade 1
Ottawa acetabular dysplasia classification	Anterior dysplasia = 1(6.7%)
	Posterior dysplasia = 5(33.3%)
	Global dysplasia = 9(60%)
Pre-PAO LCEA°	17 (-6.5 – 31)
Post-PAO LCEA°	31.9 (25.1 – 43)
Pre-PAO Tönnis angle°	11.4 (-6 – 36.4)
Post-PAO Tönnis angle°	4.3 (0 – 12.9)
Pre-Alpha angle°	> 55: 33% (5/15) with a mean of 63.2 (57–80) < 55: 67%(10/15) with a mean of 47.7(40–53)
Post-Alpha angle°	One hip with LCP at 80 degrees 14/15 < 55 with mean of 47.0(40–53)

### Operative findings and procedures

Labral damage was found in all the patients. Four hips (27%) required a FOCP for cam lesion with impingement. None of the patients required acetabuloplasty or microfracture. Operative findings and procedures are summarized in Table 3.

**Table 3:** Surgical findings and procedures of the cohort

Labral tear	15 (100%)
Labral debridement	8 (53%)
Labral repair	7 (47%)
Cartilage damage per Beck	1: 5 (33%)
	2: 1 (7%)
	3: 0
	4: 4 (27%)
	5: 5 (33%)
Adhesiolysis	2 (13%)
FOCP	4 (27%)

### Failure – conversion to hip replacement

Four hips (27%) were converted to a hip replacement at a median time of 1.8 years (0.5 – 3.2) after hip arthroscopy and 6.5 years (4–11) after PAO. Patients converted to hip replacement were significantly older (*p* = 0.01), had a lower post-PAO LCEA(*p* = 0.01) and a higher post PAO Tönnis angle (*p* = 0.02). Other factors were not significant. Predictors of failure are summarized in Table 4.

**Table 4:** Demographics and radiographic predictors of failure (conversion to hip replacement)

Predictors of failure		Conversion to hip replacement	*p*-value
Demographic findings	Gender (% Male)	Yes: 1/4 (25%)	0.42
		No: 1/11 (9%)	
	Age at time of hip arthroscopy	Yes: 38.9	**0.01**
		No: 26.6	
	BMI (kg/m^2^)	Yes: 26.4	0.75
		No: 26.6	
	Time from PAO to hip arthroscopy (years)	Yes: 4.6	0.65
		No: 3.6	
	Previous ipsilateral hip surgery	Yes: 1/4 (25%)	0.93
		No: 3/11 (27%)	
Radiographic findings	Pre PAO LCE°	Yes: 12	0.28
		No: 24	
	Pre PAO Tönnis angle°	Yes: 24	0.10
		No: 7	
	Post PAO LCE°	Yes: 27	**0.01**
		No: 40	
	Post PAO Tönnis angle°	Yes: 7	**0.02**
		No: -1	
	Ottawa classification	Yes: 1 posterior and 3 global uncoverage	0.71
		No: 1 anterior, 4 posterior and 6 global uncoverage	
	Tönnis classification	0: 3/12 (25%)	0.77
		1: 1/3 (33%)	
Surgical findings and procedures	Beck chondral damage	Yes: 1 grade 4 and 3 grade 5	0.17
		No: 5 grade 1, 1 grade 2, 3 grade 4 and 2 grade 5	
	Labral debridement only	Yes: 3 hips	0.31
		No: 5 hips	
	Labral repair	Yes: 1 hip	0.31
		No: 5 hips	
	FOCP	Yes: 1 hip	0.42
		No: 1 hip	
	Adhesiolysis	Yes: 1 hip	0.93
		No: 3 hips	

### PROMs

PROMs were collected at the last follow-up with a median of of 4 years ± 3.5 after hip arthroscopy (1 – 12) for all patients that were not converted to hip replacement.

**Fig. 2:**
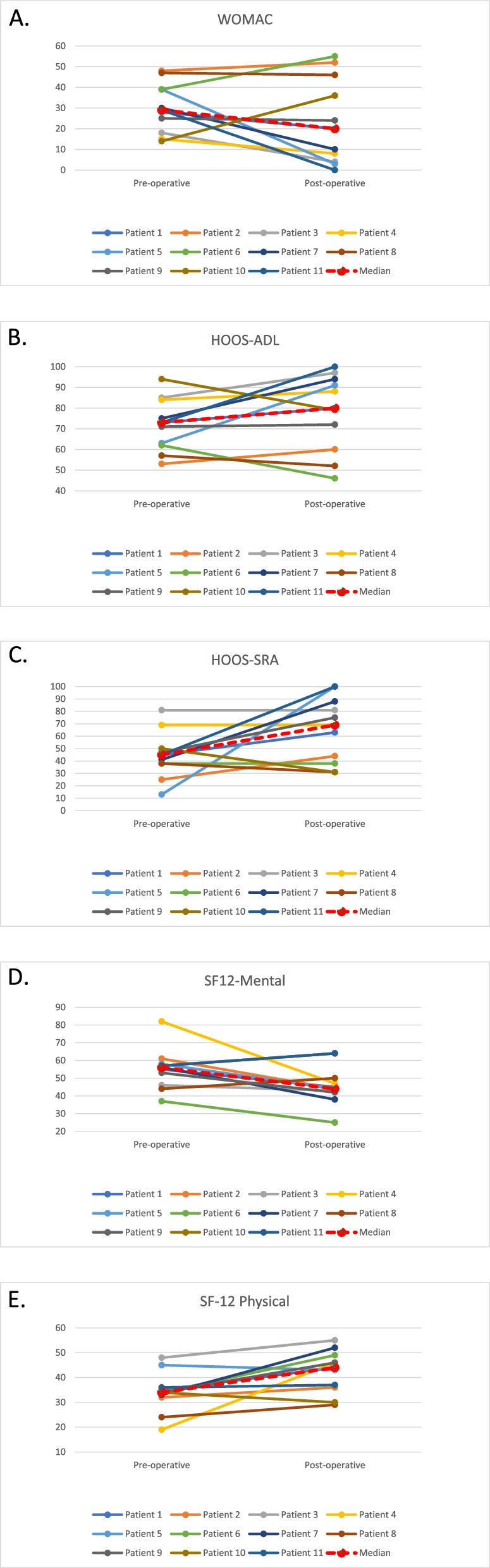
Pre and post-operative PROMs for all patients not converted to hip replacement with median range. **A**. WOMAC; **B**. HOOS-ADL; **C**. HOOS-SRA; **D**. SF12-Mental; **E**. SF-12 Physical

There was no significant improvement in any of the PROMs collected after hip arthroscopy. Three and 5 hips respectively reached the MCID and PASS for HOOS-ADL while 2 and 5 hips respectively reached the MCID and PASS for HOOS-SRA (Table 5 and Fig. 2).

**Table 5:** PROMs pre and post hip arthroscopy

PROMs	Pre-arthroscopy	Post-arthroscopy	Change	p
WOMAC total,; median(range)	29 (13—48)	20 (3 – 55)	-1 (-36—+ 22)	0.86
HOOS-ADL,; median(range)	73 (53 – 94)	80 (46 – 100)	+ 4 (-16—+ 27)	0.87
MCID	2 hips	3 hips		
PASS		5 hips		
HOOS-SRA; median(range)	45 (13 – 81)	69 (31 – 100)	+ 1 (-18—+ 87)	0.46
MCID	2 hips	2 hips		
PASS		5 hips		
SF-12 physical; median(range)	36 (19 – 51)	44 (29 – 55)	+ 6 (-5—+ 27)	0.12
SF-12 mental; median(range)	56 (37 – 82)	44 (25 – 64)	-7 (-35—+ 6)	0.25

## Discussion

The main finding of this study is that hip arthroscopy after PAO did not significantly improve PROMs. PAO has demonstrated to be the treatment of choice with optimal long-term outcomes [4, 23–25]. However, our understanding and treatment of hip dysplasia has evolved tremendously in the last decade with the advent of hip arthroscopy and minimally invasive surgery [26], plus the fact that acetabular dysplasia is accompanied by intra-articular lesions [8], some authors have advocated for isolated arthroscopic management of labral tears in the presence of acetabular dysplasia. In a recent systematic review by Yeung et al. [27] that analyzed 889 patients with acetabular dysplasia treated by hip arthroscopy alone, authors performed 27% of labral repair, 25% of FOCP, and 15% of capsular plication and closure. The majority of the studies in this systematic review showed improvement in outcome measures post-operatively however the reoperation rate was as high as 14.1% with 9.6% being converted to hip replacement at a mean follow-up of 32.2 months. Similarly, Chahabarbakhshi et al. [28] found that the improvement in PROMs for patients who underwent arthroscopy for dysplasia alone was less than in the nondysplastic control group. However, in highlighting reoperations after arthroscopy in the management acetabular dysplasia it is equally important to do the same after PAO.

Our study reports a hip arthroscopy reoperation rate of 7.4% at a mean time of 3.9 years after PAO for acetabular dysplasia. This rate is in line with the literature which reports and incidence from 2.5% to 27% [29–31]. All of the patients that underwent hip arthroscopy post PAO had persistent pain and a poor functional outcome i.e. their PROMs score were inferior to our overall results [32]. According to Hartig-Andreasen et al [30], the risks factors of needing a hip arthroscopy after PAO are preoperative borderline dysplasia, acetabular retroversion and complete labral detachment. In contrast in our study we didn’t find any particular type of dysplasia pattern at greater risk [15]. Nassif et al [31] reported that subsequent hip arthroscopy after PAO was 8.3% if patients had a PAO alone versus 2.5% for patients who had a combined PAO and arthrotomy at a mean follow-up of 2.8 years perhaps demonstrating that treatment of intra-articular lesions during PAO may decrease the need for secondary arthroscopy. Beaulé et al [33] did find that the femoral head asphericity was a risk factor for poorer scores on PROMs after PAO, perhaps by leaving intra-articular damage untreated. We found that in our cohort all patients had a labral tear, 60% had an advanced chondral damage (Beck ≥ 4),13% had adhesions and 27% had femoral asphericity. In his review of 17 hip arthroscopies after PAO (9 of which had arthrotomy during PAO), Cvetanovich [29] reported 81% of labral tears, 75% of advanced chondral damage and 43% of cam impingement which is globally in agreement with our operative findings, even though they also report 37% of pincer impingement as well as one patient with a torn ligamentum teres. These findings may provide some guidance as to which patients undergoing PAO may benefit from adjunct arthroscopy i.e. patients with femoral asphericity with alpha angle > 55 degrees. Now one might argue that since all patients had a labral tear it might be best to arthroscopy all patients undergoing PAO, but the overall percentage undergoing hip arthroscopy is low, and the majority had significant arthritic changes hence the need for a prospective randomized control trial looking at this critical question.

In our study, although some patients may have experienced subjective improvement, PROMs were not statistically improved overall in the cohort, and 27% were ultimately converted to hip replacement. Patients who were older and had more pre and post-PAO uncoverage (lower LCEA and higher Tönnis angle) were more likely to fail and be converted to hip replacement. These risks factors of failure after PAO are already known from several long term studies: Wells et al. [5] reported a 3.5 increased rate of failure in patients older than 25 years in their review of 133 hips at 18 years of follow-up. Ziran et al. [4] reported a 10-year survival of 93.3% for patients aged 20 years versus 63.2% for patients aged 50 years, in their study of 302 PAOs while Lerch et al. [25] reported a 4.3 fold increase of failure for patients older than 40 in their 30 years follow up study of 75 PAOs. Advanced age is therefore clearly a known risk factor for failure after PAO, and it is likely that in these patients the intra-articular degenerative lesions are too advanced to expect any benefit from hip arthroscopy after PAO and the preferred solution would be a hip replacement. Post-PAO acetabular uncoverage has also been shown to be a predictor of failure in several medium and long-term studies [3, 34–37]. Perhaps in these patients, bony correction may have been suboptimal and isolated correction of intra-articular lesions does not change the chondral degeneration process because the loads are still not optimally shared in the acetabulum. The patients who could expect an improvement with a hip arthroscopy after PAO of their subjective result and the survival of their native hip would therefore be young patients with optimal bone correction.

Thus, young patients, provided that the bone correction is optimal, could expect to benefit from arthroscopic treatment of their acetabular dysplasia after PAO. Based on this observation, it is also possible to ask whether these patients could benefit from a combined hip arthroscopy during the PAO and then avoid the need of another procedure after the index PAO? Maldonado et al. [38] reports the results of 16 patients undergoing PAO combined with hip arthroscopy, and at 5 years of follow-up, significant improvement in PROMs as well as the absence of osteoarthritic progression or conversion to hip replacement were observed. Kim et al. [7] also found a significant improvement in PROMs in their study of 38 hips treated by concomitant hip arthroscopy + periacetabular rotational osteotomy at a mean follow-up of 74 months.

Performing PAO immediately after hip arthroscopy can be more complicated, especially due to fluid extravasation, which makes tissue dissection during PAO more challenging. However, according to Sabbag et al. [39], who reviewed 243 PAO combined with hip arthroscopy, the complication rate at 3 years follow-up was only 3% and comparable to the complication rate of isolated PAO. Thus, it would appear that performing hip arthroscopy at the same time as PAO is an effective and low risk technique. However, to our knowledge, there is no randomized controlled study comparing PAO alone to PAO combined with hip arthroscopy. Only a reliable study like this one could confirm that arthroscopic treatment of intra-articular lesions in these dysplastic patients could improve their postoperative outcome.

The strengths of our study are that it is a prospective data collection study and we used multiple validated functional hip outcome scores. However, it has limitations: it is a single-center, non-randomized study with a small number of patients, and the results are probably difficult to extrapolate. Moreover, post-operative PROMs were collected only for hips that were not converted to hip replacement; and at the final follow-up point and not a fixed point. This could result in a bias in PROMs collection. Nevertheless, we believe that the results of our study, in particular the high rate of conversion to hip replacement (27%) and the lack of improvement in PROMs, should lead each surgeon to carefully consider the indication for hip arthroscopy after a PAO that has not improved the patient. Our experience would be to select for this indication young patients, without osteoarthritis, with optimal bone correction and proven intra-articular lesions.

## Conclusion

Our study reports a hip arthroscopy reoperation rate after PAO of 7.4%. All 3 types of dysplasia (uncovered anteriorly, posteriorly or globally) were present in our cohort showing that wherever the uncoverage there may be intra-articular lesions such as labral lesions, chondral lesions or femoral head asphericity and because 27% of patients eventually converted to hip replacement and PROMs were not significantly improved by hip arthroscopy this procedure should be approached with some caution.

## Data Availability

The datasets used and/or analyzed during the current study are available from the corresponding author on reasonable request.
